# Robot Assisted Laparoscopic Adrenalectomy: Does Size Matter?

**DOI:** 10.7759/cureus.9887

**Published:** 2020-08-20

**Authors:** Narasimhan Ragavan, Nivash Selvaraj, Deepak Raghavan, Ramesh Kamalakannan, Thirumalai Ganesan Govindaswamy, Arun Kumar Balakrishnan, Nitesh Jain

**Affiliations:** 1 Urology, Apollo Hospitals, Chennai, IND

**Keywords:** robotic adrenalectomy, tumor size, outcomes, minimally invasive surgery

## Abstract

Objective: Open adrenalectomy (OA) is considered to be the standard care for large adrenal tumors. Minimally invasive surgery (MIS) using laparoscopic technique is considered for many patients in the modern era. Robot assisted laparoscopic adrenalectomy (RALA) can be an extremely useful tool which will negate the disadvantage of laparoscopic method. The aim of the present study is to determine whether adrenal tumor size and laterality have an impact on patients undergoing RALA with respect to perioperative and postoperative outcomes.

Methods: During the study period, 38 patients who underwent RALA in a tertiary care center were considered for retrospectively analysis. The study populations were subdivided into distinctive groups based on the tumor size (<5 cm and ≥5 cm, <8 cm and ≥8 cm), and side (right and left side). For all the subgroups, perioperative and postoperative outcomes were analyzed. Perioperative and postoperative outcomes were assessed between patient groups, group a) <5 cm and ≥5 cm tumor, group b) <8 cm and ≥8 cm, and group c) laterality (right vs left).

Results: None of the patients showed any differences. In the current study, the conversion rate, readmission, and mortality were not observed. No major complications were noted.

Conclusion: RALA appears to be an extremely viable alternative to MIS using laparoscopic technique. The operative time, console time, blood loss, complication rates, and stay were extremely minimal irrespective of the size or laterality of the adrenal tumor.

## Introduction

Adrenalectomy is the standard operation for the adrenal tumors. Traditionally, adrenalectomies are done by open method especially for tumors more than 5 cm in view of the possibility of malignancy [1]. Numerous studies have verified the efficacy, safety, and feasibility of a laparoscopic approach [2-3]. Presently, laparoscopic adrenalectomy (LA) is viewed as the favored procedure of choice for adrenal tumors. Robotic technology with its advantages including a magnified three-dimensional view, endowrist technology, better ergonomics, better camera control, and reduced surgeons’ fatigability provides better alternative to laparoscopic technology. Recently, many studies as well as meta-analyses demonstrated robotic adrenalectomy (RA) supremacy over open adrenalectomy (OA) and LA [4-6].The disadvantages of RALA include higher cost and accessibility which prevented it from becoming worldwide acceptance for adrenalectomy. However, a tumor size of more than 5 cm is still believed to be a contraindication for minimally invasive procedures and the standard of care seems to be an open approach. Gryn et al. reported that tumors larger than 5 cm have unfavorable surgical outcome, positive margin, and complications [7]. In the present study, we share the experience of 38 patients undergoing RALA for various adrenal lesions.

## Materials and methods

During the observation period between January 2013 and March 2020, a total of 38 patients underwent RALA at our center. The perioperative and postoperative data were collected and analyzed. Adrenalectomies done for the suspected metastatic disease, bilateral adrenalectomies as well as adrenalectomy with concomitant procedures such as nephrectomies were excluded from the study. The outcomes were compared after dividing the study populations based on the size cut off value of a) 5 cm and b) 8 cm. Similar analyses were done to assess the differences between the study population based on laterality of the surgery. Some 5 cm was chosen as the tumor cut off value, which has been mentioned in most of the literature [7-9]. All patients underwent detailed preoperative workup to asses fitness for surgery as well as the functionality of the adrenal tumors. Radiological assessment was done in all patients by using contrast enhanced CT abdomen for assessing the tumor size as well as the nature of the tumor. The measured outcomes include tumor size, operative time, open conversion rate, estimated blood loss, postoperative complications, and duration of hospital stay.

Statistical analysis

 The comparison of study populations was done by using Microsoft Excel TM and SPSS version 20.0. Continuous variables were represented as mean ± standard deviations (SDs). As the number of patients in each subgroup was less than 30, the authors used nonparametric test to do intergroup comparison. Kruskal-Wallis test was used. Differences were considered to be statistically significant if p-value < 0.05.

## Results

A total of 38 RALA were performed by experienced robotic surgeons. Out of 38, 21(55%) were male and 17(45%) were female. The mean age of the patient was 46.5 years. The laterality of the tumor as noted includes right side n=22(58%) and left side n=16(42%). The patient characteristics, perioperative and postoperative outcomes, and histopathological diagnosis were shown in Table 1. 

**Table 1:** Patient demographic profile and perioperative outcome parameters (N=38). SD, standard deviation; EBL, estimated blood loss

Age mean ± SD (years)	46.5 ± 11.3 (18-64)
Sex Male ,% Female, %	21 (55%) 17 (45%)
Side Right, % Left, %	22 (58%) 16 (42%)
Mean tumor size ±SD, (range) cm	5.94 ± 3.2 (2-15)
Mean total operative duration ± SD, (range) mins	89.6 ± 8.1 (75-110)
Mean EBL ± SD (range) mL	54.4 ± 16.8 (35-105)
Mean console time ± SD, (range) min	43.5 ± 8.7 (33-65)
Conversion rate, %	0
Blood transfusion, %	0
Mean hospital stay ± SD, (range) days	2.7 ± 2.0 (1-12)
Histopathlogical diagnosis	Numbers
Cortical adenoma	13
Myelolipoma	12
Pheochromocytoma	6
Lymphagioma	2
Ganglioneuroma	1
Hemangioma	1
Tuberculosis	1
Adrenal cyst	2

The mean ± SD total operative time was 89.6 ± 8.1 and the mean (range) console time was 43.5 ± 8.7 min (33-65). The mean ± SD estimated blood loss was 54 ± 16.8. Moreover, no patient required blood transfusion and no patients had open conversion. The ERAS protocol was followed in all patients. 

 None of the perioperative and postoperative outcomes which were assessed between groups reached statistical significance in patients who underwent RALA are shown in Tables 2-3 with regard to tumor size cut off values of 5 cm and 8 cm. The documented blood loss between the laterality of the tumors (left vs right) showed a statistical significance of p-value (0.03) (Table 4). However, the authors believe that the overall blood loss itself was extremely low for this observation to be of any significant value. There was no observed mortality in the study population. Postoperative complications were assessed by Clavian Dindo grading. No major complications were encountered in our study.

**Table 2:** Perioperative and postoperative outcome by tumor size. IQR, interquartile range

Outcomes	<8 cm median and IQR	≥8 cm median and IQR	p value
N	29	9	
Total operative time (min)	87 and (85,95)	85 and (85,96)	0.38
Console time (min)	40 and (35,50)	40 and (38,42)	0.83
Blood loss (mL)	52 and (45,60)	45 and (45,55)	0.60
Post op complication%	0(%)	0(%)	
Conversion to open%	0%	0%	
Length of hospital stay	3 and (2,4)	1 and (2,1)	0.03
Readmission%	0%	0%	

**Table 3:** Perioperative and postoperative outcome by tumor side. IQR, interquartile range

Outcomes	Left median and IQR	Right median and IQR	p value
N	16	22	
Total operative time (min)	90 and (84,95)	85 and (85,94)	0.79
Console time (min)	45 and (37,55)	40 and (36,45)	0.24
Blood loss (mL)	55 and (49,61)	45 and (41,56)	0.03
Post op complication%	0%	0%	
Conversion to open%	0%	0%	
Length of hospital stay	3 and (2,4)	2 and (1,3)	0.06
Readmission	0	0	

 

**Table 4:** Perioperative and postoperative outcome by tumor size. IQR, interquartile range

Outcomes	<5 cm median and IQR	≥5 cm median and IQR	p value
N	16	22	
Total operative time (min)	85 and (85,91)	88 and (85,96)	0.09
Console time (min)	39 and (35,50)	40 and (38,53)	0.75
Blood loss (mL)	45 and (44,59)	54 and (45,60)	0.44
Post op complication%	0%	0%	
Conversion to open%	0%	0%	
Length of hospital stay	3 and (2,4)	2 and (1,3)	0.09
Readmission%	0%	0%	

## Discussion

Adrenal tumors are diagnosed both on incidental basis or when they have functional elements of secreting tumors. The functional (secreting) tumors present with hypertension or other features which are identified during evaluation [10]. The size of the adrenal tumors especially in incidentally diagnosed tumor does have an impact in decision making with regard to the choice for approach in adrenal surgery. The size more than 5 cm is generally suggestive of possible malignancy and hence open approach is recommended. Minimally invasive surgery (MIS) for adrenalectomy has been in practice for the last many years. Although no prospective randomized trials are there comparing LA technique with OA, LA is regarded as the suitable approach for most surgical disorders of adrenal [11]. During the era of MIS, RALA as a part of it has been available as an alternative to laparoscopic approach. Horgan et al. performed first reported use of robot in performing bilateral adrenalectomy [12]. However, there is always concern about the cost and value of RA compared with laparoscopic method. The present study compares the perioperative and postoperative outcomes in 38 patients who underwent RA with respect to tumor size and laterality.

The tumor size is an important consideration during adrenal surgery. The capsule of the adrenal is not strong and hence manipulation can potentially rupture the tumor. The size more than 5 cm with a capsular rupture, potentially possess oncological risk during an operation. Laparoscopic equipments with its innate limitations in movements as well as its reach to the interior parts of the abdomen, possess a risk of capsular rupture especially in large tumors and in obese patients. Authors believe that the robotic technology aids the handling of the tumor efficiently without causing capsular rupture. We also believe that the principle of adrenal surgery to control the adrenal vein as a first step of the surgery is well achieved with robotic technology. To corroborate one of the authors (NR) has published a case report of giant adrenal adenoma of size 15 cm which was excised efficiently by using robotic technology [13]. Many comparative studies have demonstrated the outcomes between RAs and LAs in terms of shorter operative time, lower morbidity, and decreased conversions and concluded robotic approach has better outcomes [14].

 In our current series, all RALAs were done without capsular rupture .This was in concordance with publish literature by Morelli et al. and eo et al about the feasibility of robotic adrenalectomies in larger tumors [15-16]. However, the experience of the surgeon plays a major role in the management of larger tumors.

 A series of 33 patients who underwent LAs reported by Walz et al. showed tumor with larger size (>6 cm), had longer operative time (mean ± SD, 110 ± 63 min), had greater blood loss, and had increased conversion rate comparing with smaller size tumor (<6 cm) [17]. Castillo et al. [18] showed a significant difference in operative time and blood loss in a series of 227 patients who were divided into three groups based on the tumor size (<6 cm, 6-7.9 cm, and >8 cm) and reported that tumor more than 8 cm had larger operative time range (65-120 min) and blood loss range of 50-475 mL.

 In the current study, the above observations have been largely negated and we believe it is because of utilization of the robotic technology. This was in concordance with Quadri et al. [19]. The length of hospital stay was higher in patients with tumor size <8 cm which was found to be related to logistic and social reasons.

 The laterality posseses its own difficulty for adrenalectomy. Right sided adrenalectomies include anatomical retrocaval location and short adrenal vein for the right adrenal gland. Moreover, on the left side, lot more mobilization and proximity of pancreas are issues. Rieder et al. reported right adrenalectomies have lesser operative time and blood loss compared with left adrenalectomies [20]. However, the present study showed no statistical difference in total operative time, console time, and postoperative complications with respect to tumor side, except blood loss, albeit less than 100 mL, which was found be statistically significant (p value 0.03).

The lack of availability of robotic technology could be a limitation for this procedure to be available to a wider population. The institution does one of the highest numbers of robotic surgeries in the country and the authors are very well-experienced surgeons. All of the authors had done more than 20 robotic surgeries before performing RA. RA is found to be about 10% costlier than LA in our institution. The authors strongly believe that in those patients with very large adrenal tumors especially with multi-organ involvement requiring multiorgan resection, which are best done with open method. 

In the above study, the subgroup analysis done on the patients divided between the laterality and size did not any yield any significant differences. The limitations of the study are retrospective study design, smaller size, and interpersonal variation in surgical technique by three different surgeons who have wide experience in robotic surgery. However, multicenter randomized studies are required to find out the role of robotics in adrenal surgery.

## Conclusions

Robotic assisted laparoscopic adrenalectomy is a safe method for performing minimally invasive adrenal surgeries. Thus, utilizing robotic technology negates any concerns regarding the size and laterality of the tumor. We believe that this should be the standard of care for minimally invasive adrenalectomy.
